# Pharmacochaperoning of the ER-retained A_1_ adenosine receptor

**DOI:** 10.1186/2050-6511-13-S1-A63

**Published:** 2012-09-17

**Authors:** Justyna Kusek, Christian W Gruber, Christian Nanoff, Michael Freissmuth

**Affiliations:** 1 Institute of Pharmacology, Center for Physiology and Pharmacology, Medical University of Vienna, 1090 Vienna, Austria

## Background

The A_1_ adenosine receptor is a member of the rhodopsin-related subfamily of GPCRs. Point mutations in the conserved NPxxY(x)5,6F motif at the junction of helix 7 and the C-terminus disrupt surface targeting of the receptor and result in its intracellular retention. This trafficking arrest can be overcome by addition of receptor ligands (pharmacochaperoning) that stabilize the receptor fold and thus promote surface expression. The mutants serve as a tool to explore a ramification of the retaliatory metabolite complex: hypoxia leads to intracellular accumulation of adenosine (by breakdown of ATP and by inhibition of adenosine kinase). Intracellular and extracellular adenosine levels are in equilibrium because of the action of the equilibrative nucleoside transporters. Extracellular adenosine dampens cellular metabolism by acting on inhibitory A_1_ adenosine receptors and thus counteracts the impact of hypoxia. If adenosine also pharmacochaperoned A_1_ adenosine receptors during hypoxia, it would enhance its effectiveness as a protective agent.

## Methods

We created cell lines that stably expressed A_1_ receptor Y288A with either a C-terminal YFP or an N-terminal FLAG-epitope fused to a streptactin peptide. These cell lines were incubated with a combination of inhibitors to test whether manipulations of intracellular adenosine levels increased the accumulation of the receptor. The A_1_ antagonist DPCPX was used as an internal control. To mimic hypoxia, the cells were incubated for 24 hours under 5% O_2_ content conditions. Radioligand binding and Western blotting was performed to determine the level of mature, binding competent receptors.

## Results

Inhibition of adenosine kinase, adenosine deaminase and equilibrative nucleoside transporters enhanced the accumulation of binding competent receptors. The action of these inhibitors was as effective as DPCPX in pharmacochaperoning the receptor. The receptors acquired a mature glycosylation status indicative of export from the ER and delivery to the Golgi, and they reached the cell surface. Moreover, the action of the enzyme inhibitor cocktail could also be elicited by hypoxia.

## Conclusions

Accumulation of intracellular adenosine elicits a pharmacochaperoning effect. Accordingly, the retaliatory metabolite concept may be extended to include a pharmacochaperoning (”physiochaperoning”) action of adenosine.

